# Effects of aortic irregularities on blood flow

**DOI:** 10.1007/s10237-015-0692-y

**Published:** 2015-06-25

**Authors:** Lisa Prahl Wittberg, Stevin van Wyk, Laszlo Fuchs, Ephraim Gutmark, Philippe Backeljauw, Iris Gutmark-Little

**Affiliations:** Linné FLOW Center, KTH Mechanics, 10044 Stockholm, Sweden; University of Cincinnati, Cincinnati, OH 45221 USA; Cincinnati Children’s Hospital, Cincinnati, OH 45229 USA

**Keywords:** Blood flow, Non-Newtonian, Numerical simulation, Aortic arch, Red blood cell distribution

## Abstract

Anatomic aortic anomalies are seen in many medical conditions and are known to cause disturbances in blood flow. Turner syndrome (TS) is a genetic disorder occurring only in females where cardiovascular anomalies, particularly of the aorta, are frequently encountered. In this study, numerical simulations are applied to investigate the flow characteristics in four TS patient- related aortic arches (a normal geometry, dilatation, coarctation and elongation of the transverse aorta). The Quemada viscosity model was applied to account for the non-Newtonian behavior of blood. The blood is treated as a mixture consisting of water and red blood cells (RBC) where the RBCs are modeled as a convected scalar. The results show clear geometry effects where the flow structures and RBC distribution are significantly different between the aortas. Transitional flow is observed as a jet is formed due to a constriction in the descending aorta for the coarctation case. RBC dilution is found to vary between the aortas, influencing the WSS. Moreover, the local variations in RBC volume fraction may induce large viscosity variations, stressing the importance of accounting for the non-Newtonian effects.

## Introduction

The circulatory system distributes oxygenated blood and nutrients to all parts of the body. Blood flows from the heart into the ascending aorta and then to the arch where three branches (the brachiocephalic artery, the left common carotid and left subclavian branch) supply blood to the upper part of the body. The remaining majority of the blood flow moves into the descending aorta to be distributed to the lower parts of the body. Changes in anatomy can result in abnormal blood flow pattern, which may lead to alteration of the forces acting on the vessel wall. This may also significantly change the distribution of red blood cells (RBCs). Anatomic vascular anomalies that may occur are changes in the shape of the arch, including changes in the aortic diameter or lengthening of certain portions of the arch. The aortic valve may also consist of two instead of three cusps that may cause the valves not to close or open properly, modifying the flow pattern as the blood enters the ascending aorta. These patterns of anomalies are often observed in Turner syndrome (TS) patients.

TS is a genetic disorder occurring in 1:20,000 female live births. Cardiovascular diseases are common in this patient group. The most common defect in this patient population is characterized by an increase in the distance between the left common carotid and subclavian arteries that may include a flattening and kinking of the arch. This defect, described as an elongated transverse aorta (ETA), is found in approximately $49\,\%$ of adult TS (Ho et al. 2004; Gutmark-Little and Backeljauw 2013). Bicuspid aortic valve, occurring in approximately $30\,\%$ of TS patients, is the second most common cardiovascular anomaly in TS (Gutmark-Little and Backeljauw 2013; Kim et al. 2011). Found in around $27\,\%$ of TS population, aortic dilatation, i.e., widening of aorta, is another common defect (Gutmark-Little and Backeljauw 2013). Aortic coarctation, a narrowing of the aortic diameter commonly found directly after the left subclavian artery and just before the descending aorta, appears in 8–16 % of young and adult TS patients (Gutmark-Little and Backeljauw 2013; Ho et al. 2004). Another defect found with increasing frequency in TS is partial anomalous pulmonary return (Bondy 2008a). The risk of aortic dissection is increased in TS patients, a risk that is assessed by the aortic size index, defined as the ratio of the ascending aortic diameter to the body size area. Risk of dissection is thought to be increased when this number is greater than 2.5 $\hbox {cm/m}^2$ (Bondy 2008a, b). According to Kim et al. (2011) and Matura et al. (2007), ETA is often found in combination with coarctation, bicuspid aortic valve and dilatation, and may be a contributing factor to the increased risk of aortic dissection in TS patients as compared to normal population. However, aortic dissection has also been described in TS patients without a malformed aortic valve, coarctation or other cardiac defects (Bondy 2008b).

The underlying reason for the cardiovascular defects found in TS along with the impact of these anomalies on the pathology of the disease is unknown. However, changes in flow patterns due to aortic anomalies as are found in TS in regions that are subjected to high velocities and shear stresses may have clinical implications. This in turn motivates further studies investigating the connection of changes in aortic anatomy, blood flow pattern and systemic blood pressure over time (Ho et al. 2004).

In recent years, the impact of hemodynamic patterns on the development of arteriosclerosis has received increased attention (Chen and Lu 2006; Evegren et al. 2011; Lantz and Karlsson 2012; Liu et al. 2011; Morbiducci et al. 2011; Wyk et al. 2013). In particular, the wall shear stresses (WSS) along with its spatial and temporal gradients have been considered. Various WSS measures such as average wall shear stress, oscillatory shear index and relative residence time applied near the wall have been presented (Ku et al. 1985; Lei et al. 2001; Soulis et al. 2007, 2008). A low time-average wall shear stress combined with a high oscillatory shear index has been reported to correlate with sites prone to develop atherosclerosis (Gambillara et al. 2005; Ku et al. 1985; Zarins et al. 1983). More recently, Wyk et al. (2013) found that time-average wall shear stress gradients were located at plaque-prone regions. However, the oscillatory shear index and relative residence time may also appear at locations that are at low risk of developing atherosclerosis (Wyk et al. 2014). All these studies consider blood as a continuum where the bulk properties of the fluid have been set by either assuming a Newtonian fluid or applying a non-Newtonian viscosity model. Often, a Newtonian fluid is assumed when studying complex geometries. However, neglecting the non-Newtonian effects that blood exhibits leads to significant differences in the flow field and in WSS (Wyk et al. 2013). This conclusion is also supported by Karimi et al. (2014), who showed that at peak systole there is a clear effect of blood rheology on wall shear stress, especially in the brachiocephalic and carotid branches. Karimi et al. (2014) numerically investigated the difference between nine non-Newtonian blood models applied to a healthy aortic arch concluding that all non-Newtonian blood models may be used except the Cross model.

Existing data are inconsistent regarding whether aortic flow is transitional or fully turbulent (Lantz et al. 2012; Stalder et al. 2011; Stein and Sabbah 1976). However, fully developed turbulence is rarely observed in healthy humans (Barbee 2002). In the case of turbulence, a heart murmur will be detected and in a healthy heart there will therefore not be any generation of noise (Sabbah and Stein 1976). Still, it is common to assume the flow in large healthy arteries to be turbulent. The argument for this assumption is that the flow behaves similar to steady flow in a straight pipe and that the Reynolds number is large enough to lead to transition to turbulence. In addition, arterial flows are subject to relatively high amplitude perturbations of different scales as the blood passes through the left ventricle of the heart, the aortic valve and the aortic root. With such strong perturbations, transition to turbulence would be much faster than in a pipe under laboratory conditions. Another complication regarding the use of Reynolds number for prediction of whether the blood is turbulent is the non-Newtonian behavior of blood. Stalder et al. (2011) argued that although flow instabilities are observed in healthy aortas of young males and females, the flow is not fully turbulent. The computed peak Reynolds number, i.e., the maximum Reynolds number detected during the cardiac cycle, in their study corresponded to $Re_\mathrm{peak} = \hbox {3350--4500}$ using constant density and viscosity of $\rho =1055$ $\hbox {kg/m}^3$ and $\mu =4.6\,\hbox {mPa}\,\hbox {s}$, respectively. Peacock et al. (1998) predicted that the critical peak Reynolds number is within the range 5500–9800 using an empirical equation obtained from an investigation of a pulsating flow in a straight pipe. Prior studies that have used MRI to characterize flow of malformed aortic arches include Markl et al. (2007), who reported changes in the local flow in a patient with stenoses in the subclavian branch. Keshavarz-Motamed et al. (2014) studied the flow in an aortic arch with coarctation and bicuspid valve using Particle Image Velocimetry (PIV), using a Newtonian fluid mixture of glycerol and water. With the exception of these two studies, results of detailed flow visualizations of malformed aortic arches are scarce.

The main objective of this work is to study simulated flow characteristics found in the aortic arch of TS and how they are affected by anatomic anomalies. Fluid flow in four different aortic arches from TS has been investigated. A normal aorta of TS was compared with TS with ETA, coarctation and dilated ascending aorta. A two-phase mixture model is applied to resolve the time evolution of the RBCs in the flow domain. The time-dependent flow field is fully resolved using the non-Newtonian Quemada model (Quemada 1977, 1978) to describe the viscosity of blood. The distribution of RBCs throughout the aortic arch and the related flow patterns are investigated in addition to the properties of turbulence that may exist during systole. The results reveal a clear geometry effect, leading to a redistribution of the RBCs for the malformed aortas as compared to the normal TS geometry, emphasizing the importance of viscosity modeling. Moreover, a fully turbulent flow is not observed for any of the four geometries investigated.

## Numerical method

The blood is assumed to be a non-Newtonian incompressible fluid described by the conservation of mass and momentum, supplemented by a constitutive relation describing the rheological properties of blood. The governing equations are expressed by:

1 $$\begin{aligned}&\frac{\partial \rho }{\partial t} + \frac{\partial }{\partial x_i}(\rho u_i) = 0, \end{aligned}$$

2 $$\begin{aligned}&\rho \frac{\partial }{\partial t}(u_i) + \rho \frac{\partial }{\partial x_j}(u_i u_j) = -\frac{\partial p}{\partial x_i} + \frac{\partial }{\partial x_{j}} \left( \mu \frac{\partial u_{i}}{\partial x_{j}}\right) \end{aligned}$$

3 $$\begin{aligned}&\frac{\partial H}{\partial t} = D_H \frac{\partial ^2 H}{\partial x_j \partial x_j} - \frac{\partial }{\partial x_j}(H u_j), \end{aligned}$$

where $\rho$ is the mixture density of the blood consisting of blood plasma and RBCs, *H* is the RBC volume fraction and $D_H$ is the effective mass diffusivity of the RBCs. The RBCs are modeled as a convective scalar for which time evolution is handled through the mass transport equation (Eq. 3). The effective mass diffusivity of RBCs, $D_H$, is assumed to include all forces except the convective force acting on the RBCs. Based on Wyk et al. (2013), the mass diffusivity applied in this work corresponds to a Schmidt number ($Sc = \mu / (\rho D_H)$) of 100, i.e., the effect of diffusivity is minor compared to the convective forces. The mixture density is represented by a function of the RBC and plasma densities:

4 $$\begin{aligned} \rho = \rho _H H + \rho _p(1-H), \end{aligned}$$

with $\rho _H$ (approximately 1100 $\hbox {kg/m}^3$) representing the density of the RBCs and $\rho _p$ (1025 $\hbox {kg/m}^3$) the density of the blood plasma (water plus large molecules) (Bronzino 2000; Caro et al. 2012).

Based on our previous work (Wyk et al. (2013, 2014), the Quemada viscosity model (Quemada 1977, 1978) is used to account for the non-Newtonian viscosity behavior. The Quemada model is solely dependent on the local shear rate and RBC concentration as compared to other non-Newtonian models available in the literature. Also, it is valid for a wider range of shear rates, including the lower range. Through the Quemada viscosity model, the distribution of the RBCs and their time evolution is coupled to the flow field via the dynamic shear viscosity dependency on the volume fraction of RBCs according to:

5 $$\begin{aligned} \mu = \mu _p \left( 1-\frac{k_0(H)+k_\infty (H) (\dot{\gamma }/\dot{\gamma }_C(H) )^{1/2}}{1+ (\dot{\gamma }/\dot{\gamma }_C(H) )^{1/2}}(0.5H) \right) ^{-2} \end{aligned}$$

where $\mu _p=0.00132\,\hbox {Pa}\,\hbox {s}$ (Lowe et al. 1993; Caro et al. 2012) is the plasma viscosity and parameters ${k_0}$, ${k_\infty }$ and ${\dot{\gamma }_C}$ are non-dimensional intrinsic viscosities related to the low and high shear rate ranges and the critical shear rate, respectively. Empirical correlations for each of these parameters have been investigated by Cokelet (1987) and are expressed as a function of RBC volume fraction by the following relations:

6 $$\begin{aligned}&\dot{\gamma }_C(H) = e^{\left( -6.1508+27.923H-25.6H^2+3.697H^3\right) } \end{aligned}$$

7 $$\begin{aligned}&k_0(H) = e^{\left( 3.874-10.41H+13.8H^2-6.738H^3\right) } \end{aligned}$$

8 $$\begin{aligned}&k_\infty (H) = e^{\left( 1.3435-2.803H+2.711H^2-0.6479H^2\right) } \end{aligned}$$

**Fig. 1:**
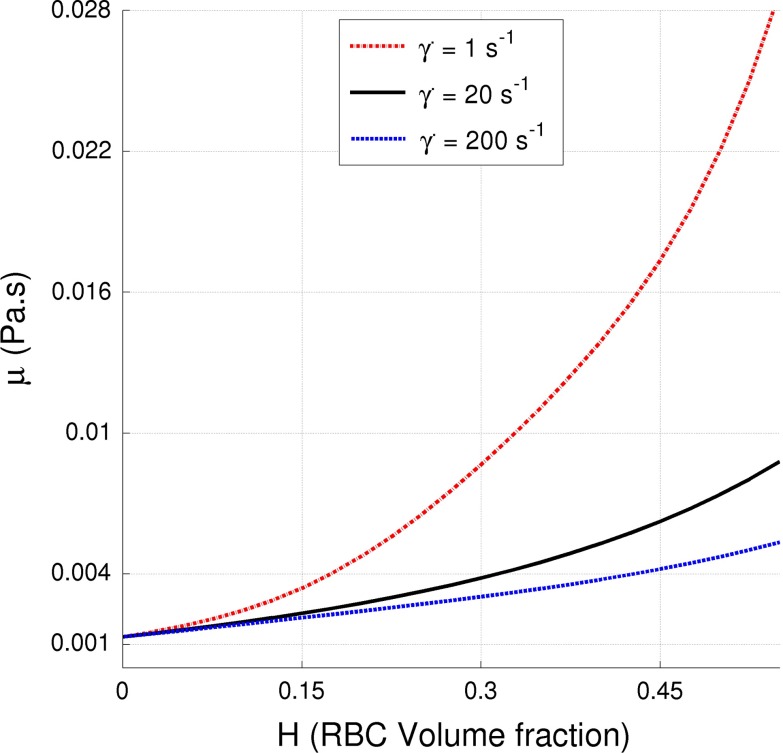
The non-Newtonian Quemada (Quemada 1977, 1978; Cokelet 1987) viscosity model dependency on RBC volume fraction at shear rates of 1, 20 and 200 $\hbox {s}^{-1}$

Figure 1 depicts the variation of viscosity per Quemada’s model relative to the RBC volume fraction, relevant to this study where three fixed shear rates are used in order to illustrate the effect of the shear thinning behavior of blood. The magnitude of the local shear stress is computed directly from the flow field and the local RBC volume fraction is computed by Eq. 3. These two parameters are then used to compute the local viscosity (Eq. 5) which is used in the momentum equation (Eq. 2) above. Moreover, there is a substantial variation of the blood viscosity, highlighting the importance of using non-Newtonian models.

The governing equations are discretized using a second-order finite-volume approximation. Coupling between velocity and pressure is handled using the PISO scheme. The time derivatives are approximated using a backward implicit discretization. In order to maintain a CFL $(=\!u \mathrm{d}t / \mathrm{d}x) <1$, an adaptive time step is applied. This is carried out in order to ensure adequate accuracy for flow transients.

The reason for choosing this approach is that the transient character of the flow implies that simplistic relationship between the flow and pressure has to be excluded. Moreover, the transitional character of the flow poses additional requirements on the modeling, excluding common approaches that rely on turbulence models such as the *k*–$\epsilon$ model.

### Model limitations

The limitations of this approach are mainly due to the assumption that the fluid behaves as a mixture and that the mixture is handled as a continuum. The physical properties, i.e., viscosity, of the mixture are given by the empirical models. The model is neither handling the motion of individual cells, nor the interaction among cells specifically. Rather, the model describes the averaged motion of the cells and the (locally) averaged viscosity of the mixture.

The effects of the blood viscosity can be modeled at different levels, depending on the assumptions made. A detailed model uses a continuum model for the plasma and a discrete approach for the cells, following each of these. This approach allows cell deformation by using a Volume of Fluid description of the cells, here used to validate the approach adopted in this study, assuming a mixture model. An intermediate approach is, for example, that of Jung and Hassanein (2008) using a multi-phase (fluid) approach where each of the constituent phases (plasma, leukocytes and RBC) has its own physical properties. These properties, together with the handling of the interaction among the phases, require further assumptions (and modeling). In that sense, the mixture model is simpler, requiring only calibration of the model applied. In this study, experimentally calibrated mixture models as published in the literature are used. It needs to be pointed out that it is not possible to accurately test the mixture model as used in this study since such experimental data considering more complex geometries are unavailable in the literature.

## Flow geometry and case setup

**Fig. 2:**
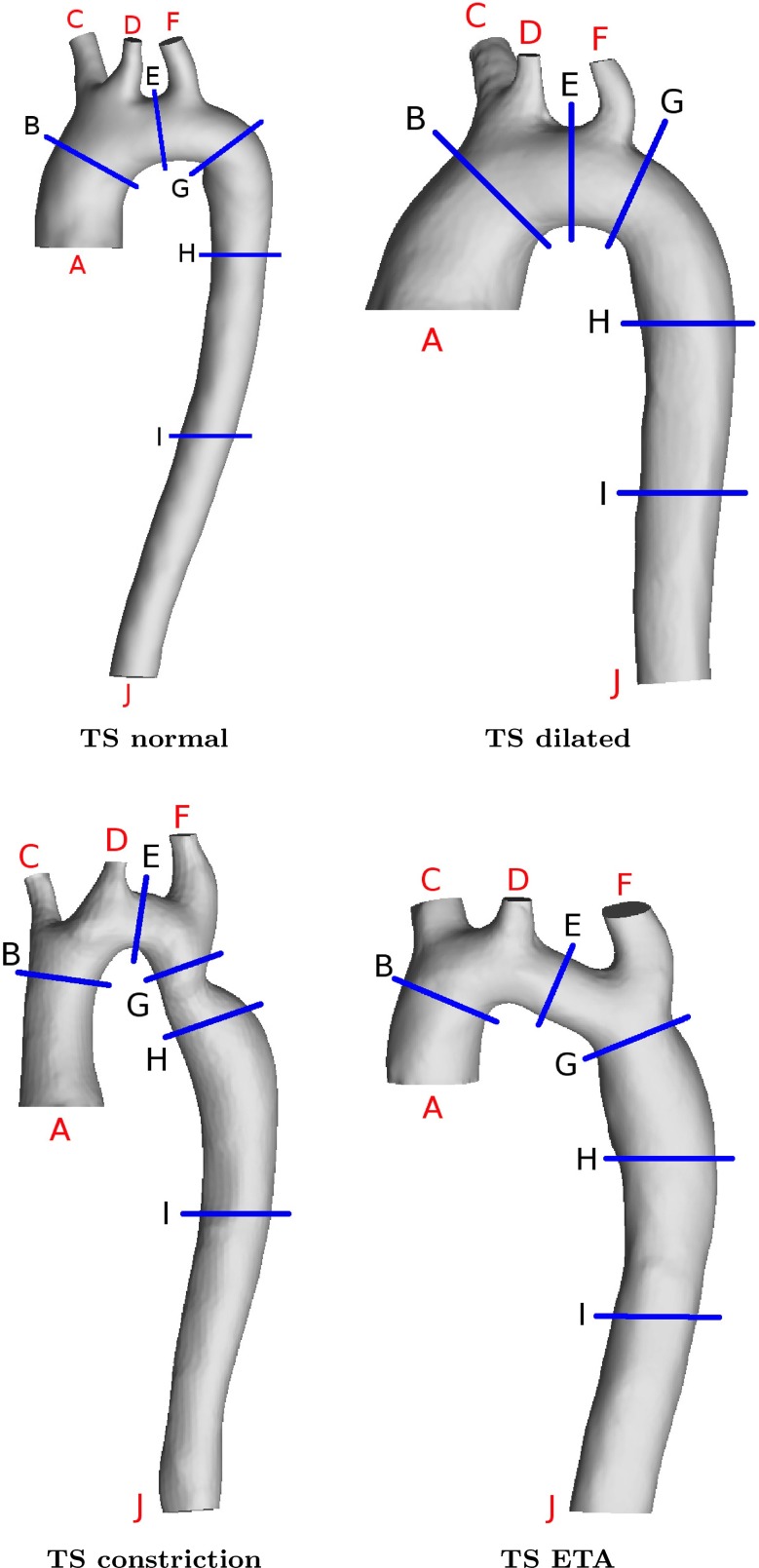
The four different patient-related aortic arch geometries studied: TS normal, TS dilated, TS constriction and TS ETA. *A* represents the inlet into the ascending aorta. The outlets *C*, *D*, *F* and *J* are the brachiocephalic branch, the left common carotid, the left subclavian branch and the outlet from the descending aorta, respectively

The four different patient-related aortic arches from TS are shown in Fig. 2. TS normal represents a healthy TS aortic arch, whereas the remaining three geometries display anatomical anomalies found in TS: dilatation of the ascending aorta (TS dilated), coarctation in the descending aorta (TS constriction) and the appearance of an elongated transverse aorta (TS ETA).

### Boundary conditions

The same inflow condition and outflow boundary conditions were applied in all cases to enable direct comparison of the flow fields due to differences in the aorta geometry in different TS patients.

A no-slip condition was imposed at the walls with zero-gradient condition for the pressure and scalar transport. The walls were assumed to be rigid. One could consider using Fluid Structure Interaction (FSI). FSI has been carried out for studies of the carotid artery (Perktold and Rappitsch 1995; Janela et al. 2010; Järvinen et al. 2008) as well as for the aortic arch (Crosetto et al. 2011; Wang and Li 2011; Reymond et al. 2013). In the aforementioned studies, non-Newtonian viscosity models are found in the studies considering the carotid artery and generalized vessels geometries. Regarding the aortic arch, studies tend to rely on the commonly accepted assumption that blood flow in large arteries can be considered Newtonian. In the study by Perktold and Rappitsch (1995), comparing the effect of rigid versus non-rigid walls, it was concluded that the variation of wall shear stress and its time derivatives was less than $10\,\%$. In an experimental study by Liu et al. (2001), the peak WSS on animal arterial models varied in the range of 4–17 %. More recently, Crosetto et al. (2011) and Reymond et al. (2013) reported the WSS to be overestimated when applying rigid walls instead of flexible walls in aortic arch simulations, in these studies represented by linear elastic models. Moreover, Wang and Li (2011) also pointed out that increased curvature of the aortic arch leads to an increase in peak stress. On the other hand, Moayeri and Zendehbudi (2003) found that the general fluid pattern did not significantly differ comparing rigid and flexible walls. A healthy young person may have around 10 % flexibility in their vessel walls. However, this changes with age. Investigating the mechanical properties of the human aorta, Roccabianca et al. (2014) reported that there is a considerable inconsistency among data found in the literature. Moreover, the material properties for blood vessels in TS are unknown although there are studies reporting arterial stiffness among patients with TS (Ostberg et al. 2004). It is also expected that the material properties vary in different TS patients, as the shape of the geometry is possibly the result of the interaction of the flow and adaptation of the tissue to the flow over a period of years.

The inlet was defined at the aortic root, just above the aortic valve. The outlet was defined as the descending aorta where it intersects the diaphragm. The branching vessels were modeled to the point where resolution of MRI did not allow adequate reconstruction.

**Fig. 3:**
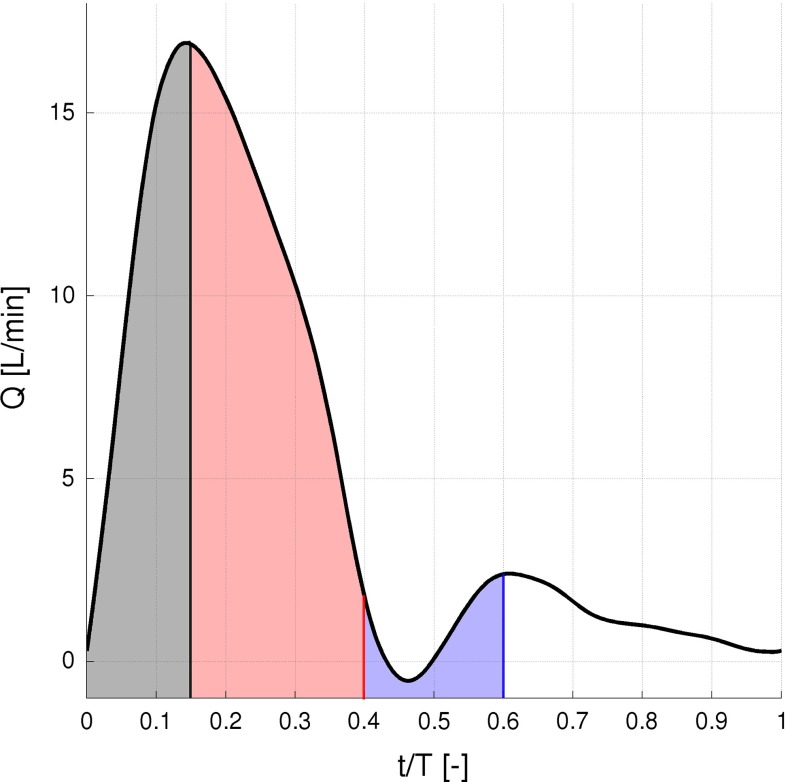
The pulsation cardiac mean flow profile at a rest pulse of 65 BPM and 5 L/min used as inflow boundary condition (Fung 2010)

**Fig. 4:**
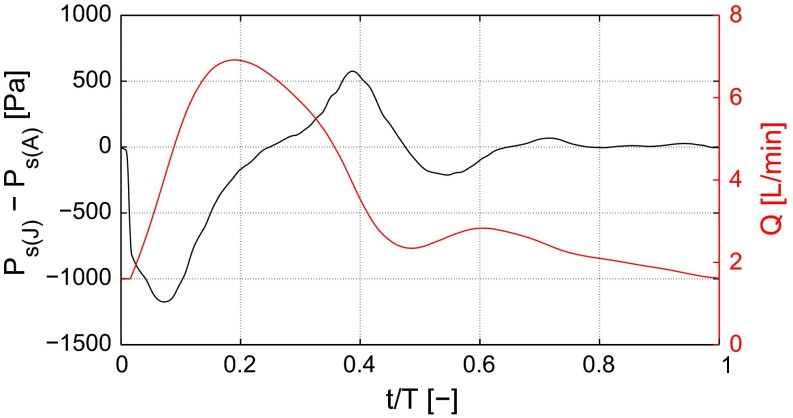
The static pressure difference between the outlet from the descending aorta (J) and the inlet (A) as well as the volume flow leaving through the descending aorta (J) for TS normal

The heart pumps approximately 5 l/min of blood at a resting pulsation frequency of about 65 beats per minute (BPM). A time-dependent inlet velocity (inlet normal component) was applied at the location indicated by (A) in Fig. 2. The temporal variation of the inlet velocity is displayed in Fig. 3 representing the typical flow in a human aorta (Fung 2010). Figure 4 depicts the pressure difference driving the flow and the volume flow for TS normal. Note the variable phase shift between pressure and flow during the cardiac cycle.

**Fig. 5:**
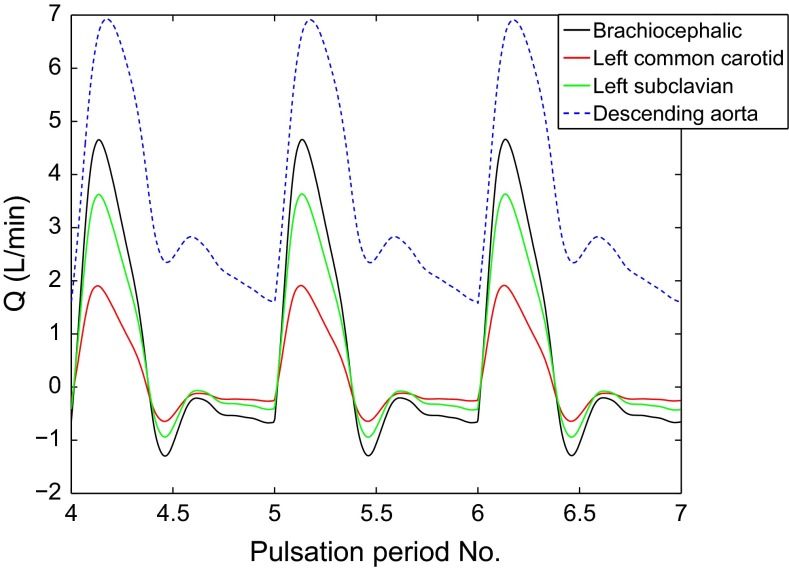
The volume flow leaving each outlet during three cardiac cycles for TS normal

In order to ensure that the computed flow was not affected by the inlet boundary condition, seven cardiac cycles were completed for each geometry. Figure 5 displays the volume flow leaving each outlet for TS normal during three cardiac cycles, showing that a periodic steady state has been reached. The results presented in this study are extracted from cycles 5, 6 and 7.

In the experimental work by Aarts et al. (1988), the distribution of RBC in large arterial vessels was close to a constant volume fraction except near the vessel wall. Subsequently, the following expression for the volume fraction RBC was used to represent the RBC distribution at the inlet (A):

9 $$\begin{aligned} H=\tilde{H} \bigl (1 + \tanh \bigl [m(r - \delta )\bigr ] \bigr ) + H_\mathrm{wall} \end{aligned}$$

where $\tilde{H}$ was chosen such that the bulk RBC volume fraction corresponds to 45 %, $H_\mathrm{wall}$ at the wall and *r* is the radial coordinate. The parameters $m\approx 5000$ and $\delta \approx 1.5$ mm are chosen to describe the RBC distribution near the wall and may vary slightly depending on the aortic inlet geometry. In this study, $H_\mathrm{wall}$ has been assigned a value of 0.25 (25 %), to impose a moderate near wall dilution. Initially, the hematocrit is set to 0.45 in the entire domain, and thereafter, the inflow boundary condition according to Eq. 9 is imposed. It should be noted that the results concerning dilution in the near wall region are sensitive to the initial settings as pointed out in Wyk et al. (2013).

For all outlets, (C, D, F and J in Fig. 2), a mean static pressure outlet condition was specified. The reason for choosing this approach, as opposed to applying a time-varying pressure wave, was to avoid additional forcing that would move the simulations toward being patient specific rather than focusing on the overall effect geometrical anomalies may have on the flow field. Moreover, the pressure variations in the chest are small. Thus, it is justified to apply a mean pressure for all outlets allowing for a pressure profile that adapts to the flow. At the beginning of each pulsation cycle, the mean static pressure, $\overline{p}$, was set to zero at all outlets. The pressure field *p* at the outlet was computed via the following condition:

10 $$\begin{aligned} p = \overline{p} - \overline{p}_\mathrm{icv} + p_\mathrm{icv}, \end{aligned}$$

where $\overline{p}_\mathrm{icv}$ represents the mean value of the *internal cell values* (icv) near the boundary and $p_\mathrm{icv}$ are the pressure field values of the cells next to the boundary. Thus, *p* is not a constant value, rather a value evolving with the flow field, while being limited by the input mean value $\overline{p} = 0$.

### Computational geometry and grid sensitivity

The 3D geometries used in the simulations were reconstructed from cardiac magnetic resonance imaging (CMR) using the medical-imaging-software MIMICS$^{\textregistered }$. Each image consisted of $256\times 256$ pixels where the pixel size was either 1.328 and 1.563 mm, depending on the scanner used. In total, 40–72 sagittal slices were used to represent the four different aortic arches, having a slice thickness of 2.2–2.8 mm. In MIMICS$^{\textregistered }$, the geometry was corrected pixel by pixel, not using a specific mask. Thereafter, the geometry was smoothed in order to be able to obtain an appropriate grid. Thus, this is not a patient-specific study in an individual sense.

**Fig. 6:**
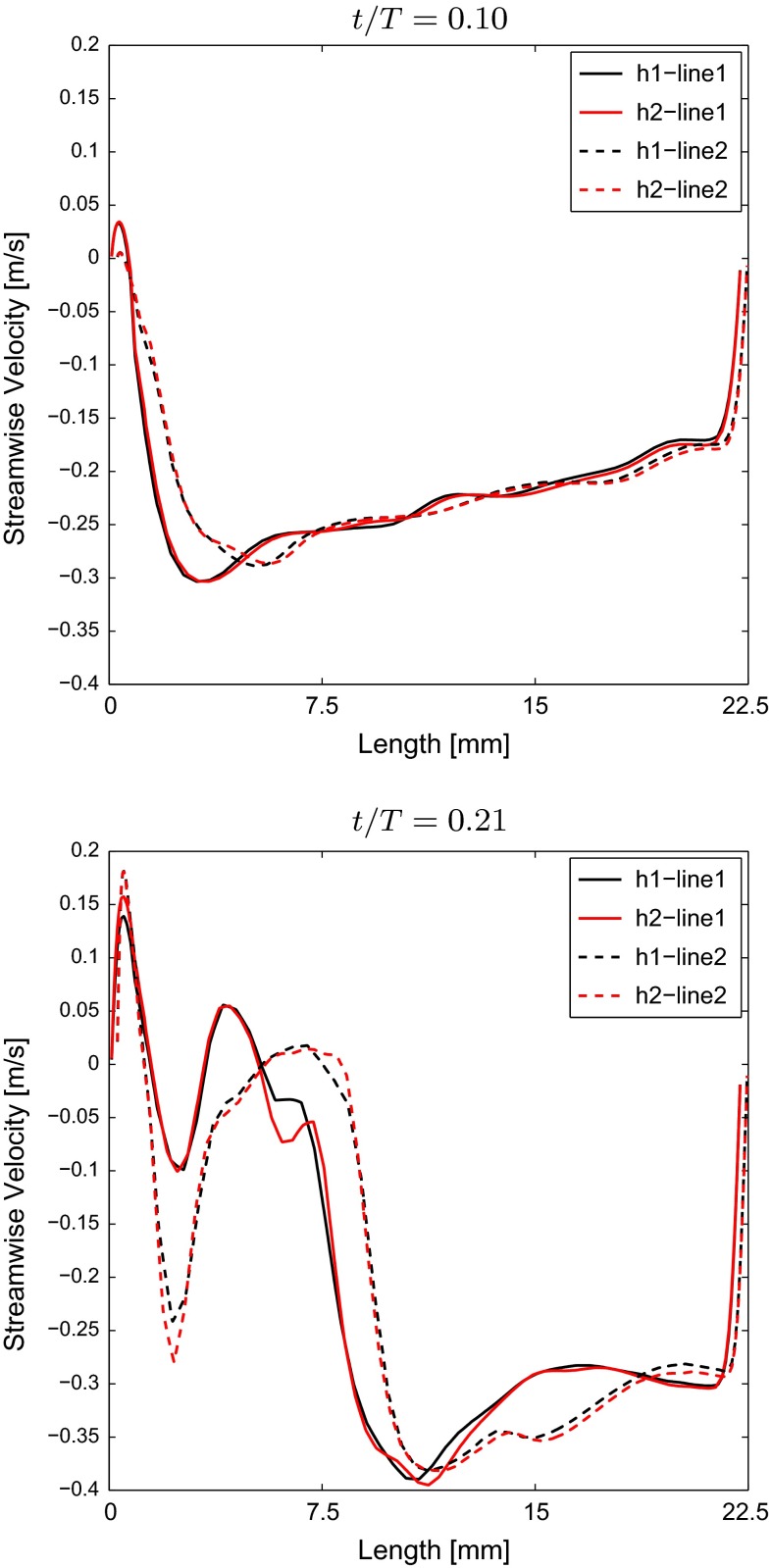
The streamwise velocity component taken across the descending aorta along the two different lines displayed in Fig. 8 at $t/T=0.1$ (*top*) and $t/T=0.21$ (*bottom*) for TS normal. The two different grids investigated are denoted *h1* and *h2*, where *h2* represents the fine grid

The computational grids were generated using Ansys$^{\textregistered }$*ICEMCFD*, consisting of a hybrid cell structure where prism layers were used at the walls in order to resolve the boundary layer, followed by a layer of tetrahedral cells acting as an interface between the prism layers and the hexahedral core. For TS normal, TS dilated, TS constriction and TS ETA, the computational grids consist of 5.8, 4.3, 5.4 and 4.7 million cells, respectively. A grid sensitivity study was performed to ensure a converged solution. Figure 6 displays the velocity profile obtained at two locations along the aorta for two grid resolutions of TS normal during the acceleration phase ($t/T=0.1$) and early deceleration ($t/T=0.21$). These lines are located at the beginning of the descending aorta where a recirculation zone develops, Fig. 8. Grid h1 consists of 5.8 million cells, and grid h2 is refined by a factor of 1.25, consisting of 7.2 million cells. The refinement is carried out by applying this factor to the number of prism cells normal to the wall, from 12 to 15 cells within 10 % of the mean radius, and the cell size of the hexahedral core. Figure 6 displays good agreement between the two grids where the estimated error according to Eq. 11 is less than 2 %, justifying the use of the relatively coarser grid.

11 $$\begin{aligned} \hbox {Error} = \frac{\sqrt{\frac{{\sum _{i=1}^N}(\phi _{1,i}-\phi _{2,i})^2}{N}}}{(\phi _{1,2,\mathrm{max}}-\phi _{1,2,\mathrm{min}})} \end{aligned}$$

Here, $\phi$ represents the property, and *N* is the number of samples.

## Results and discussion

### Flow features

**Fig. 7:**
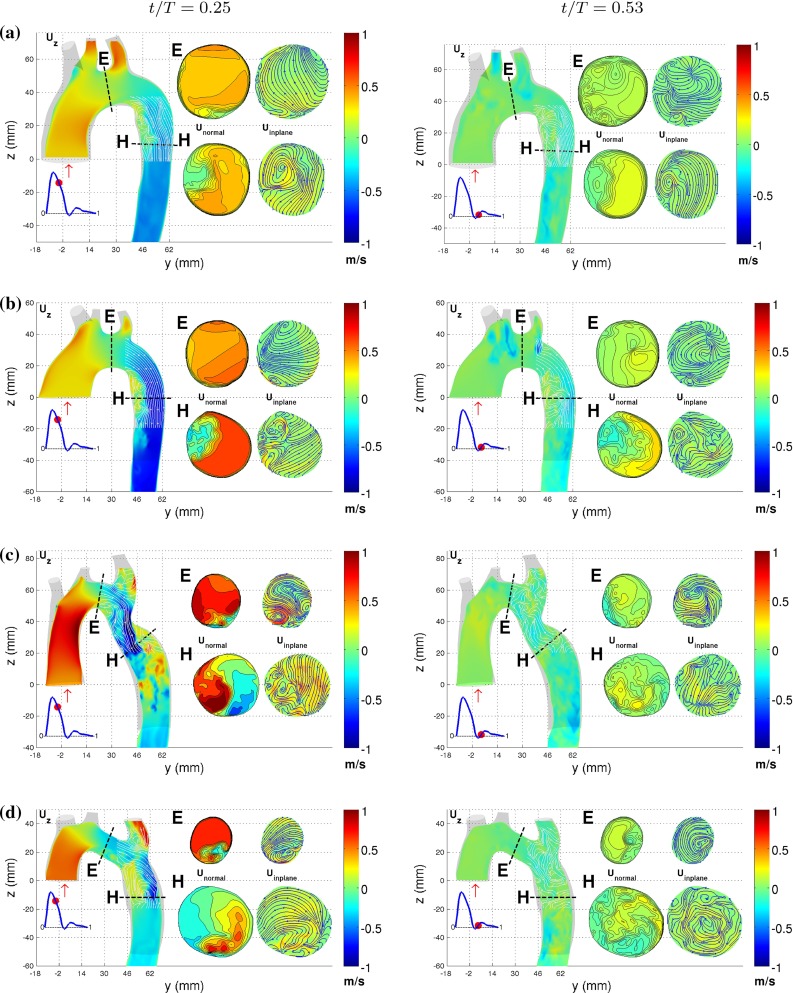
Velocity contours for **a** TS normal, **b** TS dilated, **c** TS constriction and **d** TS ETA at $t/T = 0.25$ and 0.52, displayed on the *left* and *right columns*, respectively

The computed flow field reveals a complex flow pattern, significantly different for the four aortic arches. Figure 7 displays the vertical velocity component at two points in time during the cardiac cycle, $t/T= 0.254$ (immediately following peak systole) and 0.524 (beginning of diastole). In addition, the in-plane velocity vector and the normal vector component in two cross sections (E and H) are shown. Note that these cross sections have been magnified by a factor two.

For TS normal and TS dilated, a recirculation zone develops as the flow enters the descending aorta at approximately the same time, near peak systole. This corresponds to what has been reported in the literature for a young healthy aorta assuming a Newtonian fluid flow (Shahcheraghi et al. 2002; Lantz et al. 2012), and also in the in vivo MRI study by Morbiducci et al. (2009). In our results, this recirculation zone fills half the volume of the aorta at certain points during the cardiac cycle. However, the recirculation zone is larger for TS dilated compared to TS normal, both in length and width. In TS ETA, the recirculation zone formed in the descending aorta is greater compared to TS normal and TS dilated, as depicted in cross section H in Fig. 7. In TS constriction, the stenosis causes the formation of two recirculation zones: one upstream of the stenosis, similar to the other aortic arches but less pronounced, and another immediately downstream of the stenosis. The second one is induced at the outer curvature of the descending aorta by the jet formed by the flow passing through the constriction. In TS ETA, recirculation zones are formed in the transverse aorta and at the inlet to the left subclavian branch. The latter is also clearly visible in TS constriction. Common to all four aortas is that late in the cardiac cycle, the recirculation zones and accelerated flow regions become less pronounced.

The flow of a Newtonian fluid in a curved pipe generates the streamwise Dean vortices. These vortices have been observed numerically for a pulsating flow in an asymmetric system of bifurcations and in curved arteries (Evegren et al. 2011; Chen and Lu 2006; Golbert et al. 2012). Similar vortical motion is observed in section H of TS normal, and to a lesser degree in TS dilated (Fig. 7). The vortical structures disappear during the diastolic phase of the cardiac cycle. However, these structures are not as well organized as the Dean vortices. This resembles the findings of the MRI study of a single healthy young male carried out by Morbiducci et al. (2009), reporting the flow to display bihelical flow structures that was explained to be caused by a superimposition of an axial flow over Dean-like flow structures. Outside the recirculation zone in TS normal TS dilated, the flow shows small levels of in-plane velocity motion in cross section H in Fig. 7a, b. TS ETA exhibits rotational motion that includes the entire fluid flow in cross section H in Fig. 7d which persists throughout the cardiac cycle. The flow in the descending aorta of TS constriction displays highly disorganized vortical motion in the descending aorta that also persists to the end of the cardiac cycle. Smaller localized streamwise vortices can be observed in the transverse aorta in all four cases.

**Fig. 8:**
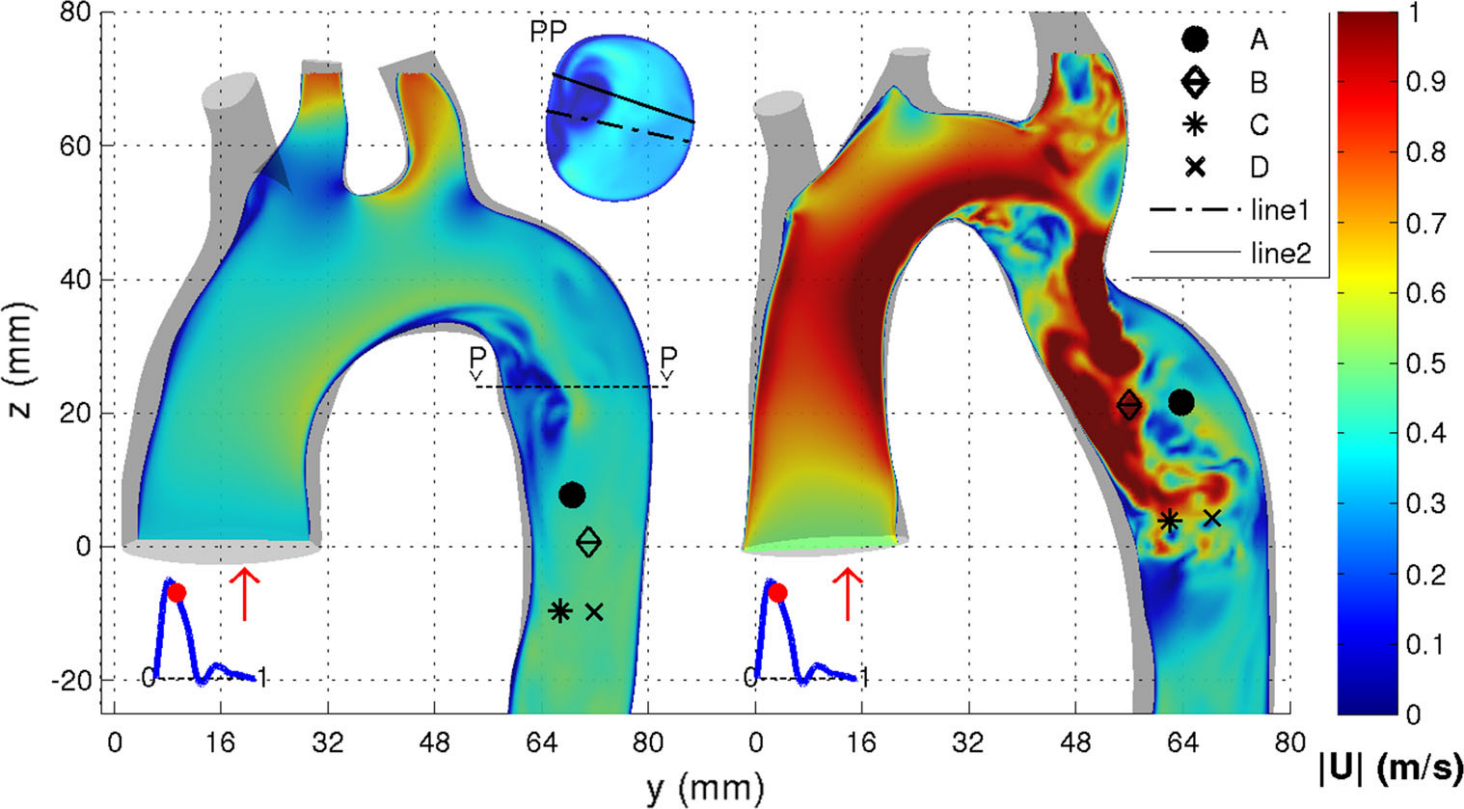
Probe locations A–D used for spectral data analysis for TS normal (*left*) and TS constriction (*right*) shown in Fig. 9. The two lines used for the grid sensitivity study for TS normal shown in Fig. 6 are also shown

#### Turbulent character of the flow

In our study, the Reynolds number is based on the computed velocity and viscosity at each time step. The viscosity is therefore not a constant. For TS normal for example, the viscosity varies between 2.5 and 4.8$\,\hbox {mPa}\,\hbox {s}$. Table 1 displays the peak Reynolds numbers as reported by Stalder et al. (2011) and the Reynolds numbers computed for the four aortas considered here at the cross sections marked in Fig. 2. The peak Reynolds numbers of TS normal are within the lower end of the range of that reported by Stalder et al. (2011). For TS dilated, TS constriction and TS ETA, the peak Reynolds numbers for certain cross sections of the aorta are greater than those of TS normal, except for locations G, H and I for TS ETA. The large variability of the Reynolds number along the aorta underscores the problem of assessing whether the blood flow is turbulent based on the Reynolds number.

**Table 1:** Peak Reynolds number for the different cross sections as indicated in Fig. 2

	B	E	G	H	I
Stalder et al. (2011)	4408	3393	3357	3860	4200
	(1036)	(709)	(590)	(543)	(447)
*TS normal*	3980	3640	2950	2670	2790
*TS dilated*	4690	4430	5070	4600	5390
*TS constriction*	6560	6650	4760	3970	3160
*TS ETA*	5560	4720	2570	1640	2130

The peak Reynolds number and its standard deviation averaged over 30 healthy young male and female volunteers reported by Stalder et al. (2011) using flow-sensitive MRI. The MRI data were obtained at locations corresponding to the positions of the planes B, E, G, H and I according to Fig. 2 used in present study

In order to determine the development of turbulence in the aortic arch, velocity data were sampled during two pulsation cycles at four different locations for the TS normal and TS constriction. These two geometries were chosen as they displayed the least (TS normal) and most (TS constriction) chaotic flow behavior among the four geometries investigated. The sampling locations, displayed in Fig. 8, were chosen in regions where the flow field structure indicated possible transition to turbulence. The sampling rate corresponded to 10,000 Hz covering 60 % of the pulsation cycle which was divided into three sampling intervals: $t/T=$ 0–0.15, 0.15–0.4 and 0.4–0.6, covering the systolic and the initial diastolic phases (Fig. 3).

**Fig. 9:**
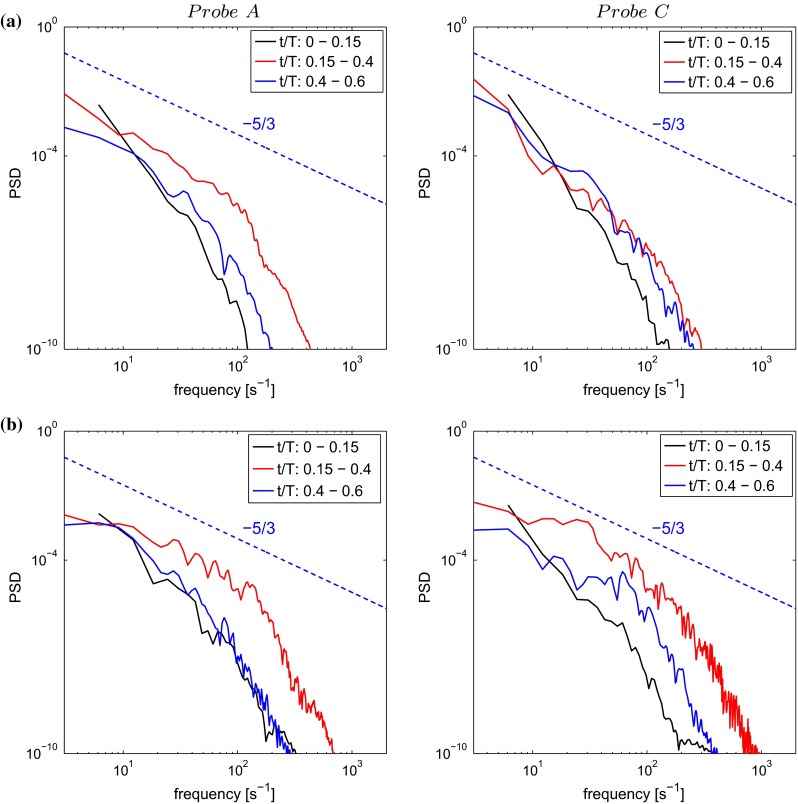
Power spectral density (PSD) of the velocity recorded at two different locations in the descending aorta (locations A and C) for **a** TS normal and **b** TS constriction. The locations of A and C are displayed in Fig. 8

Figure 9 shows the power spectra density (PSD) of probes A and C for TS normal and TS constriction. The slope of $-5/3$, characteristic of the inertial subrange of fully developed turbulent flows, is shown. The inertial subrange is observed at high Reynolds numbers, not typical to most arterial flows. The inertial subrange is not detected for TS normal, suggesting that in a healthy aorta, there is no development of turbulent length scales. However, for TS constriction, the inertial subrange is detected at all probe locations, especially immediately following peak systole ($t/T=0.15$–0.4). The wider range of turbulent scales for this case is due to the formation of a jet downstream of the constriction leading to initial development of turbulent structures. Using the constriction diameter as a characteristic length scale, the Strouhal number (dimensionless frequency) would be about 0.30, typical for jet flows. The shear-layer instability of the jet is much stronger than that of the instability induced by the centrifugal forces imposed by the curvature of the aortic arch. This jet is formed only during systole, so the turbulent burst is quite short. A frequency of 27.5–30.5 Hz (Probe A and $t/T=0.15$–0.4) within the low end of the audible range is detected for this case, which could potentially be detected by a stethoscope.

#### Mechanical losses

Anatomic arch anomalies can potentially affect the resistance of the aorta and the associated energy losses and therefore impact the work required by the heart. The energy losses within each aortic arch were estimated via:

12 $$\begin{aligned} \hbox {Loss}=\int _{0}^{T} \bigl [ (pQ)_\mathrm{in} - (pQ)_\mathrm{out} \bigr ] \mathrm{d}t \end{aligned}$$

where *p* is the total pressure (Pa), *Q* is the volumetric flow rate ($\hbox {m}^3/\hbox {s}$) and integration is carried out for a cardiac cycle period *T*.

The losses correspond to 0.12, 0.18, 0.27 and 0.09 J/cardiac cycle for TS normal, TS dilated, TS constriction and TS ETA, respectively. Thus, the work required to overcome the losses in the aortic arch is similar for TS normal and TS ETA. However, for TS dilated and TS constriction, the losses are increased by a factor 2 and 3, respectively.

**Table 2:** The total volumetric blood flow leaving each branch. Presented in percentage of what enters the aorta during a cardiac cycle

	Descending aorta [J (%)]	Brachiocephalic branch [C (%)]	L. common carotid [D (%)]	L. subclavian artery (F)
*TS normal*	70	13	5	12
*TS dilated*	87	9	2	2
*TS constriction*	62	17	12	9
*TS ETA*	50	36	8	6

### Impact on blood distribution due to anatomic anomalies

**Fig. 10:**
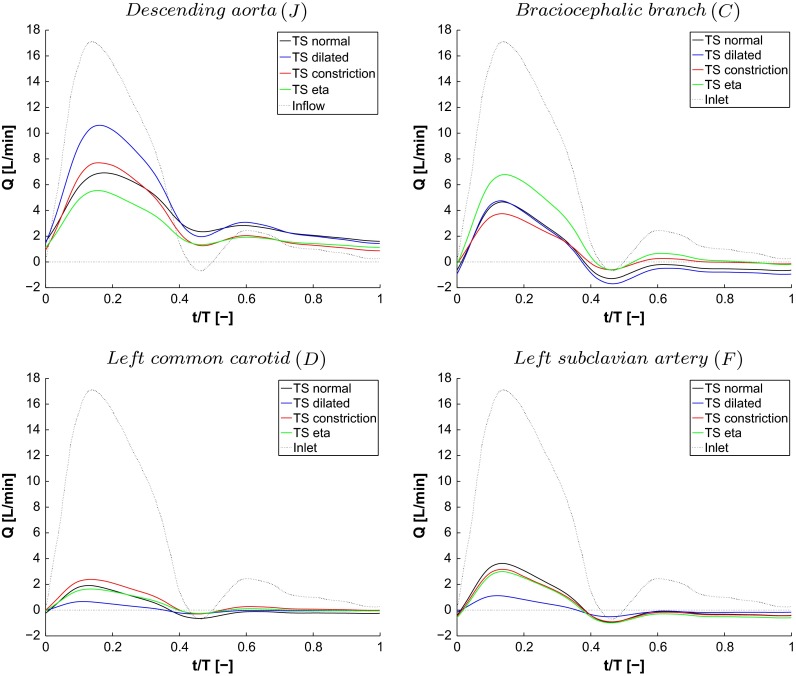
The volume flow leaving the descending aorta (A), brachiocephalic branch (C), left common carotid (D) and left subclavian artery (F) for each of the aortas investigated

As reported by Middleman (1972), about $15\,\%$ of the flow entering the ascending aorta leaves through the Brachiocephalic branch (C) and approximately $7.5\,\%$ through each of the left common carotid (D) and left subclavian branch (F). The remaining $70\,\%$ flows through the descending aorta. The volumetric blood flow leaving each branch of the four aortas during a cardiac cycle is shown in Fig. 10 and summarized in Table 2. TS normal shows similar behavior to Middleman (1972). In TS dilated, the flow leaving through the branches on the arch is decreased compared to the values by Middleman (1972), whereas the flow in the descending aorta is increased to $87\,\%$. For TS constriction and TS ETA, the flow distribution is altered to a greater extent. The flow through the brachiocephalic branch and the descending aorta corresponds to 17 and $60\,\%$, respectively, for TS constriction. For TS ETA, the corresponding volume flows are 36 and $50\,\%$. Thus, the shape of the aortic arch significantly alters the distribution of the volumetric flow through each branch during the cardiac cycle. The clinical impact of the redistribution of blood flow into the different branches is unclear, since we cannot correlate our findings to clinical data. However, an altered blood distribution through a certain vascular tree may affect the systematic pressure through the pressure regulatory sensors located in the carotid sinus and the kidneys. It is also clear that the redistribution of blood affects the wall shear stress (WSS) and its gradients in the affected vessels.

#### Blood dilution: effects on bulk viscosity

**Fig. 11:**
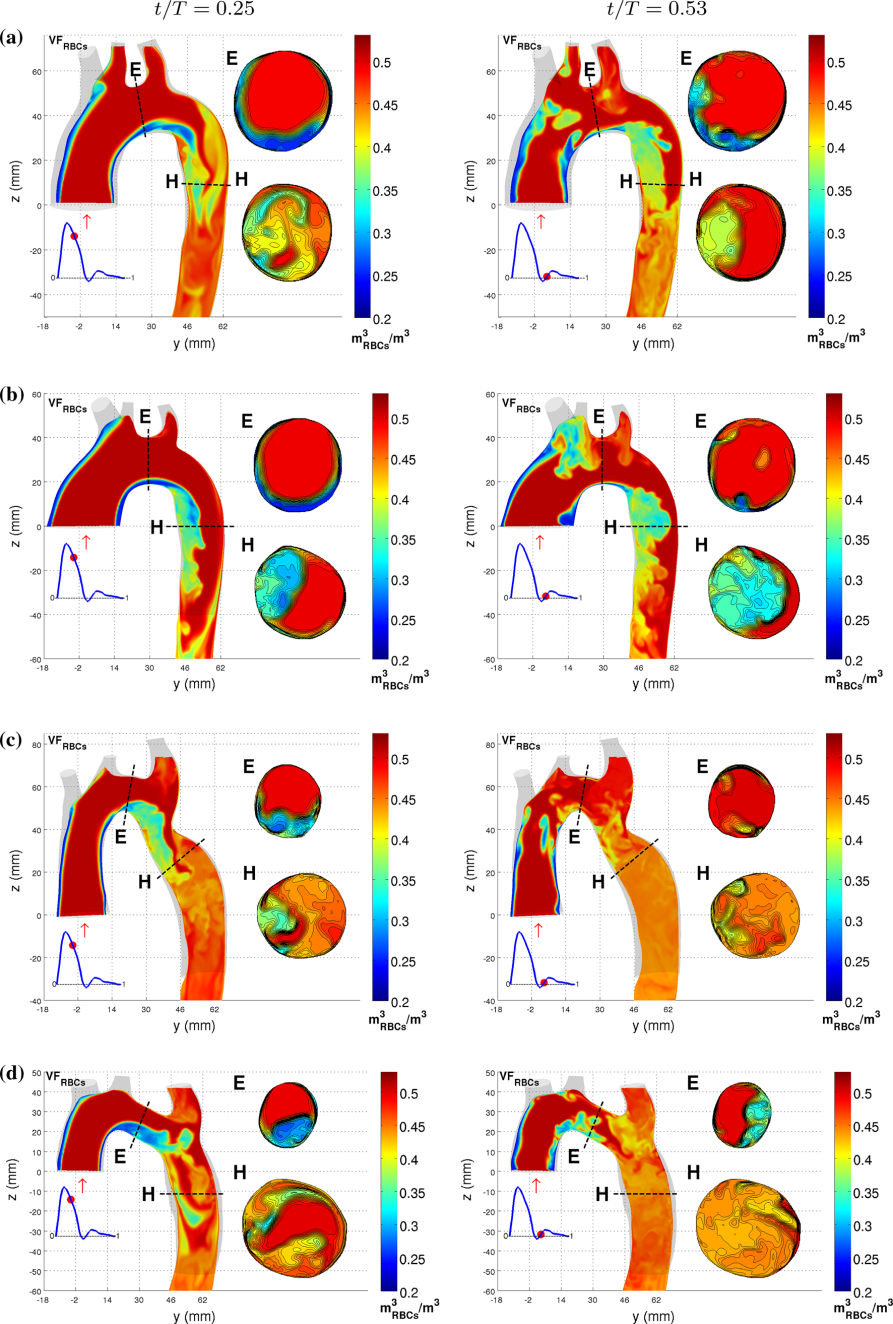
Contours of the RBC distribution for **a** TS normal, **b** TS dilated, **c** TS constriction and **d** TS ETA at $t/T = 0.25$ and 0.53, displayed on the *left* and *right columns*, respectively

The results show the distribution of RBCs in the aorta significantly differs between the four investigated geometries. In Fig. 11, the distribution of the RBCs in the aortic arch at the same point in the cardiac cycle as those shown for the velocity field in Fig. 7. Two main observations, related to the appearance of recirculation zones and the flow behavior, can be made. For TS normal and TS dilated, the volume fraction of RBCs is lowered in the recirculation zone, while the volume fraction of RBCs is slightly elevated in the flow around the recirculation zone. Also, the volume fraction of RBCs is more evenly mixed throughout the descending aorta in TS constriction and TS ETA, a feature associated with the occurrence of vortical structures in the descending aorta.

In order to estimate the dilution, i.e., the volume fraction of RBCs in the blood flow leaving the aorta through the four different outlets compared to the volume fraction of RBCs entering the aorta, the following definition has been used:

13 $$\begin{aligned} D= \frac{H_\mathrm{in}- H_\mathrm{out}}{H_\mathrm{in}} \times 100 \end{aligned}$$

where $H_\mathrm{out}$ is the volume fraction of RBCs leaving the branch of interest during the cardiac cycle and $H_\mathrm{in}$ is the volume fraction of RBCs entering the ascending aorta during the cardiac cycle, set at 45 % according to inflow boundary condition for RBC (Sect. 3.1). Table 3 presents the dilution for all branches in each aortic arch, where a positive value indicates that the flow is diluted. It is interesting to note that all aortas show no dilution for the flow exiting via the descending aorta. However, for the brachiocephalic artery, the dilution reaches values up to $10\,\%$. For TS constriction and TS ETA, elevated volume fractions of RBCs are found for the left common carotid and the left subclavian branch.

**Table 3:** Dilution (%), based on volume fraction RBC, according to Eq. 13

	Descending aorta [J (%)]	Brachiocephalic branch [C (%)]	L. common carotid [D (%)]	L. subclavian artery [F (%)]
*TS normal*	0.0	7.0	0.8	-1.4
*TS dilated*	-1.3	10.6	9.6	-0.6
*TS constriction*	-0.4	6.2	-7.0	-5.9
*TS ETA*	0.0	9.3	-3.6	-4.1

The importance of these results relates to the large variations of RBC volume fraction throughout the domain as the local volume fraction of RBCs is directly coupled to the oxygen transport capacity and the local viscosity. A change in local RBC volume fraction of $5\,\%$ may lead to an increase/decrease in local viscosity corresponding to values of $40\,\%$ depending on shear rate, Fig. 1. This in turn will directly influence the flow field and, as recently reported by Wyk et al. (2013), affect the magnitudes of the wall shear stresses (WSS) and its gradients acting on the vessel wall.

### Wall shear stress

The human arteries adapt to a WSS of approximately 1.5 Pa and will react in order to maintain this value (Glagov et al. 1988). If experiencing higher WSS, there is an elevated risk of mechanical induced blood trauma (Schima and Wieselthaler 1995). As stated in the Introduction, low and oscillatory WSS as well as elevated WSS gradients have been correlated to sites prone to develop atherosclerosis, indicators that are affected by the flow in the near wall region (Ku et al. 1985; Soulis et al. 2007, 2008; Gambillara et al. 2005; Wyk et al. 2013). Moreover, Piron et al. (2008) found that children with TS have a greater carotid intima-media thickness, a known risk factor for the development of atherosclerosis in adults. Thus, it is important to understand the effect on WSS behavior due to the geometrical anomalies found in TS.

The behavior of WSS during pulsatile flow is in the literature commonly described through the time-averaged wall shear stress (TAWSS) and the oscillatory shear index (OSI) (Chen and Lu 2006; Lantz et al. 2012; Liu et al. 2011; Morbiducci et al. 2011);

14 $$\begin{aligned} \hbox {TAWSS}= & {} \frac{1}{T} \int _0^T \hbox {WSS} \mathrm{d}t \end{aligned}$$

15 $$\begin{aligned} \hbox {OSI}= & {} 0.5 \times \Biggl [ 1- \frac{ {\bigl |\int _0^T \hbox {WSS} \mathrm{d}t\bigr |} }{\int _0^T | \hbox {WSS} | \mathrm{d}t} \Biggr ] \end{aligned}$$

In this study, the TAWSS, OSI and WSS are phase-averaged data of 3 cardiac cycles instead of a single pulse (Sect. 3.1). An $\hbox {OSI}$ of 0 indicates that the instantaneous WSS vector is aligned with the time-averaged vector during the entire cardiac cycle, whereas a highly oscillatory behavior is indicated by $\hbox {OSI}=0.5$.

**Fig. 12:**
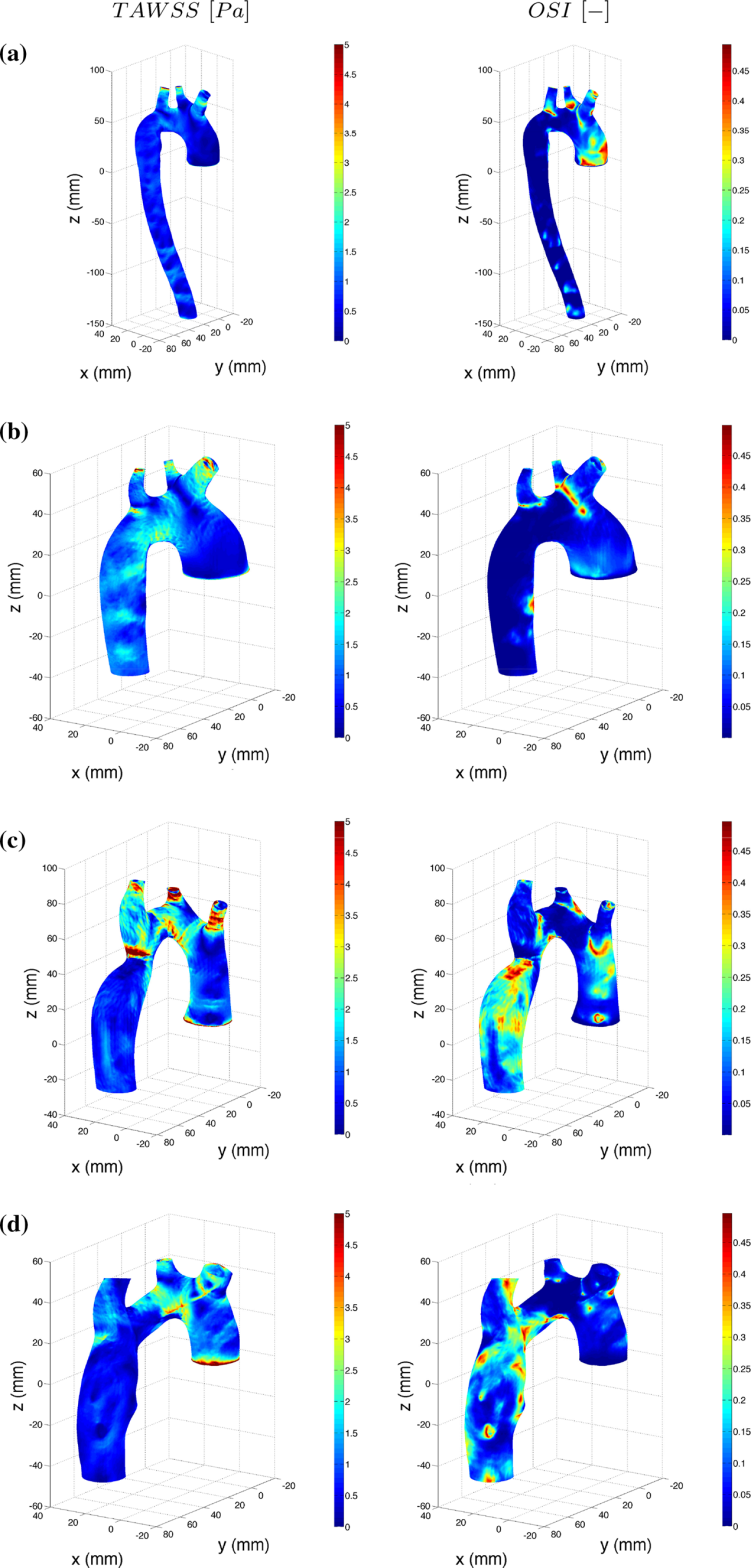
The TAWSS and OSI for **a** TS normal, **b** TS dilated, **c** TS constriction and **d** TS ETA. TAWSS and OSI are displayed on the *left* and *right columns*, respectively

Figure 12 displays the TAWSS and OSI for all four TS- related geometries on the left- and right-hand sides, respectively. Common for all geometries is that low TAWSS, i.e., $<$0.5 Pa, is found at locations where the recirculation zones are found and is thus also characterized by a high OSI. The highest TAWSS for TS normal corresponds to a value of 3 Pa and is found on the walls of the Braciocephalic branch, left common carotid and left subclavian branch, similar to TS dilated and TS ETA. However, TS dilated and TS ETA also display a high TAWSS of 5 Pa in the curve of the arch and in the elongated transverse aorta, respectively. TS constriction is subjected to the highest magnitudes of TAWSS where values around 10 Pa are found at the location of the constriction. Moreover, in TS constriction, between the inlet and the constriction, TAWSS greater than 1.5 Pa is found in the entire arch. For TS normal on the other hand, the TAWSS maintain a value around 1.5 Pa throughout the entire aortic arch including the descending aorta, a feature not observed for the three geometries displaying anomalies.

## Conclusion

The flow in four TS patient-related aortas has been investigated using a mixture model solving for the full flow field. A mixture of plasma and RBCs was introduced at the inlet of the aortic arch allowing the local density to be computed as the RBCs were convected in the flow. The local density was coupled to the Quemada viscosity model to account for the non-Newtonian behavior of blood. This is a novel approach to study these complex flows. The main findings of this work can be summarized as follows:

- The flow patterns, blood and RBC distribution into the branches reveal a clear geometry effect. In TS constriction and TS ETA, the flow is diverted to a greater extent into the branches as compared to TS normal and TS dilated.
- The flow in TS normal, and different TS variants are not fully turbulent even intermittently. Transitional flow with a limited range of scales may occur when a jet is formed, like in TS constriction.
- The flow influences the distribution of RBCs throughout the aorta and in the branches.
- In TS normal and TS dilated, the recirculation zone in the descending aorta appears as an isolated bubble with lower concentration of RBCs as compared to the surrounding flow.
- For TS constricted and TS ETA, the flow helps to mix the RBC concentration, displaying a lower, more homogeneously distributed volume fraction of RBCs.
- RBC dilution is found to vary between the geometries investigated, especially in the brachiocephalic branch, the left common carotid and the left subclavian branch, a feature that in the work by Wyk et al. (2013) was reported to influence the magnitudes of WSS and its gradients.
- The results stress the importance of viscosity modeling as the observed local variations in RBC volume fraction may induce large local variations in viscosity.
- The mechanical losses estimated in the aortic arch were of the same order of magnitude for TS normal and TS ETA, but were increased for TS dilated and TS constriction compared to TS normal.
- TS normal maintains a TAWSS around 1.5 Pa throughout the entire arch, whereas the geometries displaying anatomic anomalies are all subjected to higher values of TAWSS.
